# Artificial Intelligence Applications to Support Physical Activity, Mobility, and Fatigue Management in People with Multiple Sclerosis: A Scoping Review

**DOI:** 10.3390/healthcare14142219

**Published:** 2026-07-21

**Authors:** Pantazis Deligiannis, Iosif Alexandros Kouidis, Anastasia Theofanous, Maria Anifanti, Asterios Deligiannis, Evangelia Kouidi

**Affiliations:** 1 Microsoft Research, 14820 NE 36th St., Redmond, WA 98052, USA; pdeligia@me.com; 2 Laboratory of Sports Medicine, School of Physical Education and Sports Science, Aristotle University of Thessaloniki, Thermi, 57001 Thessaloniki, Greece; joskouidi@gmail.com (I.A.K.); manyfant@phed.auth.gr (M.A.); adeligia@phed.auth.gr (A.D.); 3 Department of Medicine, School of Medicine, European University Cyprus, Egkomi, 2404 Nicosia, Cyprus; siapafos@gmail.com

**Keywords:** multiple sclerosis, artificial intelligence, physical activity, gait, mobility, fatigue, fall risk, scoping review

## Abstract

**Background:** Physical activity plays a vital role in caring for people with multiple sclerosis (MS). Yet, challenges like fatigue, mobility impairment, fall risk, symptom fluctuation, and poor access to rehabilitation often make sustained participation difficult. Adhering to physical activity programmes is tough. Artificial intelligence (AI) may transform wearable, smartphone, clinical, and patient-reported data into interpretable information for monitoring, prediction and individualized support. **Objective:** Building on the identified need for individualized support, this scoping review mapped original AI-based studies relevant to physical activity, gait, mobility, ambulation, fatigue, fall risk, and real-world functioning in people with MS. **Methods:** The review followed PRISMA-ScR principles. Search covered records published from 1 January 2016 to 14 May 2026 across biomedical, rehabilitation, technology-oriented, trial registry, and citation-searching sources. Eligible studies included MS or MS-specific data, an explicit AI/ML or model-derived predictive analytics component, and an activity-related clinical domain. **Results:** The search identified 332 records/reports. After removing 127 duplicates, 205 records were screened, 45 full texts were assessed, and 21 original AI studies met the eligibility criteria. These addressed inertial sensor deep learning, wearable gait speed estimation, postural sway fall risk prediction, connected device prediction of fatigue and health state, smartphone-based ambulation characterization, treadmill or walkway gait classification, chair stand fall status prediction, daily life gait/turning fall prediction, fear-of-falling detection, sensor-derived fatigue prediction, mobile/wearable modelling of MS, and physical activity prediction. **Conclusions:** AI can extract clinically relevant patterns from gait, wearable, smartphone, connected device, clinical, and patient-reported data in MS. However, evidence does not yet show that AI-supported systems improve physical activity, adherence, fatigue burden, fall risk, quality of life, or functional independence. AI is therefore promising but early, and prospective, externally validated, human-supervised, fatigue-aware, safety-sensitive studies are needed to support clinically meaningful, patient-centred MS rehabilitation and activity planning.

## 1. Introduction

Multiple sclerosis (MS) is a chronic immune-mediated disease of the central nervous system and a major cause of neurological disability in young and middle-aged adults. Its clinical course is heterogeneous, and neurological impairment may affect ambulation, balance, fatigue, cognition, vision, sensation, mood, and participation in social and occupational life [1,2]. This heterogeneity is particularly important when considering physical activity, because the barriers to activity are rarely limited to muscle weakness or impaired gait alone. They frequently include fatigue, heat sensitivity, fear of falling, fluctuating symptoms, depression, reduced confidence, limited access to specialist rehabilitation, and difficulty translating clinic-based recommendations into daily life. Together, these factors may contribute to reduced physical activity, physical deconditioning, loss of functional independence, and poorer health-related quality of life, emphasizing the need for individualized, long-term, and adaptive rehabilitation strategies.

Physical activity and exercise are now considered essential components of comprehensive MS care. International recommendations support regular physical activity across the disease course. They also call for individualized adaptation to disability level, comorbidity, fatigue, and symptom fluctuation [3]. Systematic reviews and meta-analyses show that exercise can improve walking capacity, balance, fatigue, and quality of life. However, effects vary by intervention type, baseline disability, adherence, and the selected outcomes [4]. Behaviour change interventions can boost physical activity in MS. Sustained adherence remains inconsistent, which reinforces the need for personalized, scalable, and low-burden support systems [5,6].

Monitoring physical activity in MS is clinically important, because mobility, walking endurance, fatigue, fall risk, participation in daily activities, functional independence, and health-related quality of life are central to patient-centred care. Periodic clinic-based assessments may fail to capture day-to-day variability in fatigue, heat sensitivity, symptom burden, and real-world ambulation. Continuous or repeated digital monitoring may therefore help clinicians detect declining activity levels, unsafe gait patterns, fatigue-related pacing needs, fall risk behaviours, and disengagement from rehabilitation programmes. Such longitudinal monitoring may support earlier intervention, more individualized exercise prescription, and more responsive rehabilitation strategies based on real-world patient behaviour rather than isolated clinic-based assessments.

Artificial intelligence (AI) has been proposed as a clinical amplifier, not a replacement for professional judgement. In medicine, AI may help with pattern recognition, prediction, remote monitoring, risk stratification, and personalization. These uses require outputs to be transparent, valid, clinically interpretable, and embedded within proper governance [7]. Rather than replacing clinicians, AI is intended to augment clinical decision-making by assisting healthcare professionals in analyzing complex and potentially multimodal datasets. In MS, AI has mostly been used for imaging, diagnosis, prognosis, disease monitoring, disability assessment, and digital biomarkers [8]. However, the exact role of AI in supporting physical activity and real-world mobility is less clearly defined.

This distinction is central to the novelty of the present review. Several studies have examined digital health, wearable, telerehabilitation, rehabilitation technologies, mHealth or broad AI applications in MS; however, these studies address different research questions. Digital health reviews frequently include remote monitoring platforms, mobile applications, wearable devices, or telehealth interventions that do not incorporate explicit AI or machine learning methodologies. Conversely, many AI studies in MS focus on imaging, lesion segmentation, prognosis or diagnosis rather than activity-related clinical domains. Consequently, the current evidence base provides only limited insight into how AI is being used specifically to support gait, mobility, fatigue, physical activity, fall risk, rehabilitation, and real-world functional monitoring.

The novelty of this scoping review therefore lies in its AI-only focus on original studies addressing activity-relevant MS domains. By deliberately distinguishing AI-based approaches from broader digital health technologies, this review provides a more precise framework for evaluating the current contribution of AI to physical activity and mobility-related MS care. This distinction is important because including digital interventions without explicit AI-driven analysis could overestimate the current clinical maturity of the field.

The objective of this scoping review was to identify and map original AI-based studies in people with MS or using MS-specific datasets, with relevance to physical activity, gait, mobility, fatigue, fall risk, rehabilitation, and real-world functional monitoring. The review also examined AI methodologies, data sources, model development strategies, validation approaches, reported performance metrics, clinical contributions, limitations, and translational readiness. Attention was given to model generalizability, digital health equity, implementation barriers, and future AI-supported clinical applications. By synthesizing the available evidence within a clearly defined AI-only framework, this review aims to provide clinicians, rehabilitation specialists, researchers, and digital health developers with a balanced overview of the current capabilities and limitations of AI for supporting physical activity and mobility in people with MS.

## 2. Materials and Methods

### 2.1. Review Design and Rationale

This study was conducted as a scoping review because the evidence on AI applications relevant to physical activity, mobility, fatigue, fall risk, gait, rehabilitation, fall risk assessment, and real-world functioning in MS is emerging, heterogeneous, and methodologically diverse. The purpose was to map the available original AI-based evidence, describe its clinical and methodological characteristics, identify knowledge gaps, and evaluate translational readiness, rather than estimate a pooled intervention effect.

The review was informed by established scoping review methodology and reporting guidance, including Arksey and O’Malley, Levac et al., updated Joanna Briggs Institute guidance, the PRISMA extension for scoping reviews (PRISMA-ScR), and guidance on choosing between systematic and scoping review approaches [9,10,11,12,13]. Search reporting was aligned with PRISMA-S and PRESS principles [14,15]. Eligibility was framed conceptually using the Population–Concept–Context approach: the population was people with MS or MS-specific data; the concept was original AI, machine learning, deep learning, predictive analytics, or digital biomarker research; and the context included clinical, laboratory, home-based, wearable, smartphone, instrumented gait, connected device, rehabilitation or real-world monitoring settings relevant to MS care.

For this review, AI was operationally defined as computational methods capable of learning from data or generating model-derived prediction, classification, regression, clustering, or pattern recognition outputs relevant to MS-related physical activity, gait, mobility, ambulation, fatigue, fall risk, rehabilitation, or real-world functioning. Eligible methods included supervised and unsupervised machine learning, deep learning, convolutional or neural network architectures, ensemble learning techniques, regression-based predictive modelling, feature selection algorithms, and digital biomarker models when these were explicitly developed for prediction, classification, or clinically meaningful pattern recognition.

Predictive analytics was considered eligible only when the study used explicit model-derived prediction or classification relevant to MS-related physical activity, gait, mobility, fatigue, fall risk, rehabilitation, or real-world functioning. Conventional descriptive statistics, hypothesis testing procedures, correlation analyses, digital monitoring without model-derived prediction, and telerehabilitation or mHealth interventions without explicit AI/ML or predictive analytics components were not classified as AI studies. Statistical regression or association analyses used only for explanatory or hypothesis testing purposes were not considered sufficient for inclusion unless they formed part of an explicit predictive modelling framework.

Eligible AI methodologies included, but were not limited to, convolutional neural networks applied to inertial sensor gait signals, ensemble regression models using connected device data, supervised machine learning classifiers applied to treadmill or instrumented walkway gait features, interpretable deep learning models using smartphone-based ambulation data, wearable sensor-based fall status prediction, feature selection ensembles, random forest or related ensemble approaches, and multimodal predictive models integrating sensor-based, clinical, and self-reported measures.

The search covered publications from 1 January 2016 to 14 May 2026 and was conducted across biomedical, rehabilitation, technology-oriented, trial registry, and citation-searching sources. The sources included PubMed/MEDLINE, Europe PMC, IEEE Xplore, ACM Digital Library, Google Scholar, Cochrane/CENTRAL, ClinicalTrials.gov, Scopus, Web of Science, and citation list of relevant articles. This multisource approach was chosen because the review question spans neurology, rehabilitation, artificial intelligence, wearable sensing, digital mobility assessment, biomedical engineering, and computer science. All records and reports were evaluated within a single screening framework, using the same predefined eligibility criteria regardless of source.

The search strategy combined three core concepts: multiple sclerosis; artificial intelligence, machine learning, deep learning, neural networks, predictive analytics, predictive models, algorithms, or digital biomarkers; and physical-activity-related outcomes, including exercise, walking, gait, mobility, ambulation, fatigue, fall risk, telerehabilitation, and real-world functioning. These searches were adapted to the syntax of each source while preserving the same conceptual structure. Full search logic and source-specific adaptations are available in the PRISMA-ScR search documentation and the Appendix A and Appendix B.

A representative PubMed/MEDLINE search string was: (“multiple sclerosis”[Title/Abstract] OR MS[Title/Abstract]) AND (“artificial intelligence”[Title/Abstract] OR “machine learning”[Title/Abstract] OR “deep learning”[Title/Abstract] OR “neural network”[Title/Abstract] OR algorithm[Title/Abstract] OR “predictive analytics”[Title/Abstract] OR “predictive model*”[Title/Abstract] OR “digital biomarker*”[Title/Abstract]) AND (“physical activity”[Title/Abstract] OR exercise[Title/Abstract] OR walking[Title/Abstract] OR gait[Title/Abstract] OR mobility[Title/Abstract] OR ambulation[Title/Abstract] OR fatigue[Title/Abstract] OR fall*[Title/Abstract] OR rehabilitation[Title/Abstract] OR telerehabilitation[Title/Abstract] OR “real-world functioning”[Title/Abstract]).**

### 2.2. Selection Process, Data Charting, and Synthesis

Records from all sources were integrated into a single selection process and assessed using the same predefined eligibility criteria. Reference records were managed in Zotero to support duplicate checking, reference organization, and consistency of citation numbering [16]. Duplicate records were removed based on bibliographic metadata, including title, authors, year, DOI, PMID, and source, as well as other available record identifiers. Title/abstract screening and full-text eligibility assessment were performed independently by two reviewers. Disagreements or uncertain cases were resolved through discussion and, when necessary, through consultation with the principal investigator of the review until consensus was achieved. Full-text exclusions were recorded and grouped by reason.

Inter-rater agreement statistics were not calculated during the original screening process; therefore, no Cohen’s kappa value is reported. This is acknowledged as a methodological transparency limitation. Nevertheless, the review process used independent screening, predefined eligibility criteria, and consensus-based resolution of disagreements to maximize methodological consistency and reduce selection bias.

For each included study, data were charted on author/year, study design, population or data source, sample size where reported, participant characteristics including age profile where available, AI/ML method, learning strategy, feature selection or feature engineering approach, validation strategy, reported performance metrics, explainability or interpretability where reported, physical-activity-related domain, key findings, main contribution, and principal limitations. Whenever methodological details were incompletely reported in the original publication, no assumptions were made and the corresponding information was recorded as not reported.

Findings were synthesized narratively and presented in chronological order of publication, rather than by database source, publication venue, indexing status, or perceived prestige. Studies were additionally grouped by clinical application area, including gait analysis, mobility classification, fall risk or fall status prediction, fatigue modelling, ambulation monitoring, fear-of-falling detection, digital biomarker development, and real-world physical activity prediction. As this was a scoping review, formal risk-of-bias assessment was not undertaken; however, the methodological strengths and limitations of each study were critically described, with particular attention to validation approaches, performance reporting, model generalizability, reproducibility, and translational implications.

## 3. Results

### 3.1. Study Selection

**Figure 1:**
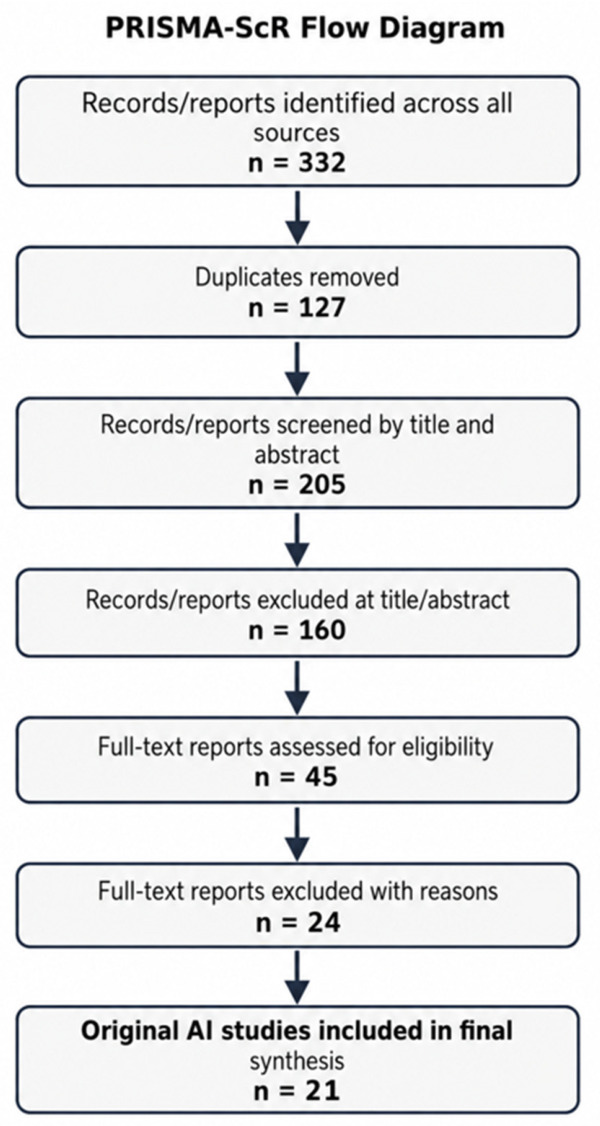
PRISMA-ScR flow diagram for the selection of original AI studies.

The multisource search identified 332 records/reports before deduplication, including records retrieved from Scopus and Web of Science. All records and reports, irrespective of source, were assessed within a single screening framework using the same predefined eligibility criteria. After removal of 127 duplicates, 205 unique records/reports were screened by title and abstract. Of these, 160 were excluded because they did not meet the population, AI/ML, study-type-, or physical-activity-relevant eligibility criteria. Forty-five full-text reports were assessed for eligibility, and 24 were excluded with reasons. Twenty-one original AI studies met the inclusion criteria and were included in the final synthesis (Figure 1).

The included studies were published between 2016 and 2025 and are presented in strict chronological order to reflect the evolution of the field, rather than database source, indexing status, publication venue, or perceived hierarchy of evidence. The final evidence base included studies of inertial sensor deep learning, wearable gait speed estimation, postural sway fall risk prediction, connected device prediction of fatigue and health state, wearable/deep learning fall risk classification, smartphone-based ambulation characterization, instrumented treadmill and walkway machine learning classification, chair stand fall status prediction, daily life gait and turning fall prediction, feature selection ensemble approaches, walking bout duration analyses, fear-of-falling detection, wearable digital biomarker fatigue prediction, mobile/wearable modelling of MS status and function, and prediction of real-world physical activity.

Most included studies evaluated AI for prediction, classification, or monitoring rather than AI-guided therapeutic interventions or behaviour change strategies. Overall, the available evidence was dominated by observational, methodological, and model development investigations, whereas prospective intervention studies and externally validated clinical AI applications remained comparatively limited.

### 3.2. Structured Categorization by Clinical Application and Data Source

The 21 included studies could be grouped into five clinically relevant application areas [17,18,19,20,21,22,23,24,25,26,27,28,29,30,31,32,33,34,35,36,37]. First, gait analysis and mobility classification included studies using inertial body sensors, skin-mounted wearable sensors, smartphone ambulation data, instrumented treadmill data, instrumented walkways, and standardized gait analysis systems to characterize MS-related gait impairment or classify MS status. Second, fall risk and fall status prediction represented the largest application area, including postural sway machine learning, wearable deep learning fall risk classification, clinical fall and injury prediction, chair stand fall status prediction, daily life gait and turning analysis, feature selection ensemble approaches, walking bout duration analyses, and fear-of-falling detection. Third, fatigue and health state prediction comprised connected device models and wearable digital biomarker approaches for estimating fatigue or health status. Fourth, real-world physical activity forecasting was represented by integrated multimodal prediction models combining clinical, sensor-based, and self-reported measures. Fifth, comparative mobility modelling studies provided additional methodological context for identifying MS-related motor signatures using walking parameters.

Overall, gait analysis, mobility assessment, and fall risk prediction constituted the predominant areas of AI research identified in this review, whereas comparatively fewer studies specifically addressed fatigue modelling or the prediction of real-world physical activity. Across these categories, the main data sources included inertial measurement units, skin-mounted sensors, wearable sensors, smartphones, connected wellness devices, instrumented treadmill systems, instrumented walkways, standardized gait analysis laboratories, postural sway measures, functional chair stand tests, daily life gait and turning signals, patient-reported outcomes, and clinical assessments.

The predominance of wearable-, smartphone-, and movement-related sensor data highlights the current emphasis on digital mobility phenotyping in people with MS. In contrast, relatively few studies integrated multimodal datasets combining clinical, behavioural, physiological, and other complementary sources of information. Despite the diversity of clinical applications, most AI models were developed using structured sensor-derived or clinically acquired datasets, reflecting the current emphasis on objective measurement and quantitative characterization of mobility-related outcomes in people with MS (Table 1).

### 3.3. AI Methodological Characteristics and Performance Reporting

Table 2 summarizes the principal methodological characteristics of the AI models included in this review, including the clinical application, sample or data source, AI/ML approach, learning strategy, validation approach, reported performance metrics, and principal methodological limitations of each study. Where methodological details were not explicitly reported or could not be reliably extracted from the original publication, this information was conservatively recorded rather than inferred to preserve methodological transparency and avoid interpretation bias.

The included studies demonstrated considerable methodological heterogeneity with respect to AI model architecture, learning strategy, validation approach, dataset characteristics, and performance reporting. This diversity reflects the evolving nature of AI research in MS and supports the use of a scoping review methodology to map, rather than quantitatively compare, the available evidence.

Across the included studies, supervised machine learning approaches predominated, whereas comparatively fewer investigations employed deep learning architectures or more advanced predictive frameworks. Internal validation strategies were reported more frequently than external validation, while prospective validation and independent replication across different populations or clinical settings were uncommon.

Performance reporting most frequently included measures of accuracy, sensitivity, specificity, area under the receiver operating characteristic curve (AUC), and prediction error, although the selection and reporting of performance metrics varied considerably between studies. Explainability or model interpretability was explicitly addressed in only a limited number of studies, and information regarding algorithm transparency, calibration, fairness, or clinical deployment was generally sparse. Overall, most AI models should currently be regarded as proof-of-concept or early translational tools rather than clinically established decision support systems.

### 3.4. Age Profile of Included Studies

Across the included studies, the available evidence mainly concerned adults with MS, most commonly young-to-middle-aged or middle-aged participants. Reported mean ages generally ranged from the mid-30s to early 50s, although some connected device and wearable sensor studies included broader adult age ranges. Where reported, study populations encompassed different levels of disability and disease duration; however, these characteristics were variably described and were not consistently incorporated into model development or validation.

None of the included studies focused specifically on pediatric, adolescent, or very elderly MS populations, and none was primarily designed to compare AI-supported physical activity, mobility, fatigue, or fall risk applications across predefined age strata. Similarly, relatively few studies evaluated model performance according to demographic or clinical subgroups, limiting conclusions regarding potential differences across patient populations.

Consequently, the current evidence base is most applicable to adult populations with MS, whereas the generalizability of existing AI models to younger, older, or clinically distinct populations remains uncertain. Future studies should evaluate AI performance across broader demographic and clinical characteristics to improve external validity and clinical applicability.

## 4. Discussion

### 4.1. Principal Findings

This scoping review identified 21 original AI studies on physical activity, mobility, gait, fatigue, fall risk, and real-world functioning in people with MS. The evidence base was particularly informative for fall risk prediction, gait analysis, and wearable/sensor-based mobility modelling. The field has progressed from inertial sensor deep learning and wearable gait speed estimation to models that use smartphones, postural sway, instrumented walkways, functional chair stand testing, daily life gait and turning, connected devices, digital biomarkers, and multimodal clinical, sensor-based, and self-reported data. The evidence indicates that AI can detect or predict functional states that are difficult to assess during periodic clinic visits. Most included studies were observational, validation, classification, or prediction studies. The most consistent contribution of AI was not autonomous exercise prescription, but the extraction of clinically meaningful patterns from complex mobility, wearable, smartphone, connected device, gait analysis, balance, fatigue, and patient-reported data. AI is currently more mature as a tool for monitoring, classification, prediction, and risk stratification than as a tested intervention for improving physical activity, adherence, fall outcomes, fatigue burden, or quality of life. These findings indicate that, at present, the principal clinical value of AI lies in supporting clinical assessment and individualized decision-making rather than replacing conventional rehabilitation strategies. AI should therefore be regarded as a clinical decision support technology that augments, rather than replaces, clinician expertise in supporting physical activity and rehabilitation in people with MS.

### 4.2. Comparative Evaluation of the Included Studies

The sequence of studies shows clear progress. Chronologically, the evidence shows a shift from controlled gait sensing toward broader functional prediction. Gong et al. introduced deep learning for extracting gait information from inertial sensors [17], and McGinnis et al. extended wearable sensor modelling toward gait speed estimation in MS-relevant mobility assessment [18]. Tong et al. advanced this by moving from lab-based assessment to longitudinal monitoring of fatigue and health state using connected devices [19]. Sun et al. added postural sway machine learning for fall risk prediction [20]. The 2021 studies strengthened the clinical relevance of AI in mobility. Meyer et al. linked wearable gait analytics to deep learning fall risk classification [21]. Creagh et al. demonstrated that smartphones could support interpretable deep learning for the characterization of remote ambulation [22]. Kaur et al. used instrumented treadmill gait dynamics and showed that ML could distinguish MS-related gait features [23]. Piryonesi et al. modelled falls and injuries using machine learning algorithms [24], while Trentzsch et al. used machine learning to identify gait parameters sensitive to subtle change in MS [25]. Together, these studies show that AI can translate raw movement, gait, and clinical data into markers of mobility impairment, fall risk, and functional change.

This evolution continued in 2022, when the two Hu studies added value by using an instrumented walkway and gait/balance data to classify MS and corroborate subjective walking and balance difficulty [26,27]. Tulipani et al. evaluated unsupervised wearable sensor chair stand testing for fall status prediction [28], while Arpan et al. used daily life gait and turning measures for fall prediction [29]. Schumann et al. applied feature selection ensemble and machine learning algorithms to gait-based fall risk detection [30]. Meyer et al. showed that walking bout duration influences fall risk classification performance in an open-source MS dataset, adding value for reproducibility and dataset-level method development [31].

Most recently, the 2024 and 2025 studies moved the field closer to real-world application. Gül et al. used walking parameters and machine learning to discriminate MS-related motor patterns in a comparative neurological context [32]. Moebus et al. identified digital biomarkers that predict daily fatigue, which is highly relevant to pacing and safe activity planning [33]. Similarly, Gashi et al. used mobile and wearable sensor data to model MS status, type, disability, and fatigue, demonstrating the potential of multimodal digital assessment [34]. Özgür et al. used machine learning to determine fall risk factor in MS [35], and Schumann et al. used machine learning to detect fear of falling during standardized gait analysis [36]. Monaghan et al. aligned most closely with physical activity promotion, as it predicted real-world physical activity using clinical, sensor-based, and self-reported data [37]. Taken together, these studies illustrate a clear methodological evolution from proof-of-concept AI models developed under controlled laboratory conditions toward increasingly sophisticated multimodal approaches capable of analyzing real-world mobility and behavioural data. Nevertheless, evidence demonstrating that AI-guided interventions translate into measurable improvements in patient-centred clinical outcomes remains limited.

### 4.3. Relationship to Digital Health, Wearables, Telerehabilitation, and Activity Behaviour Change Evidence

The AI-only evidence base is narrower than the broader digital health literature in MS. Wearable sensor reviews show rapid growth in the use of accelerometers, gyroscopes, smartphones, and other sensors to assess motor function in laboratory and free-living settings [38]. mHealth reviews show that digital interventions in MS are increasingly used for self-management, fatigue, mobility, cognition, mood, and rehabilitation support [39]. Internet-delivered, provider-supported physical activity interventions, such as BIPAMS and related trials, provide important behavioural evidence even when they do not include AI components [40].

This distinction is essential. General digital interventions should not be counted as AI studies unless they include explicit AI/ML or model-derived predictive analytics methods. However, they form the clinical ecosystem in which future AI systems are likely to operate. AI-supported physical activity promotion will likely require integrating wearable monitoring, patient-reported outcomes, fatigue-aware feedback, telerehabilitation, behaviour change techniques, and clinician oversight. AI-based telerehabilitation protocols such as PLATINUMS illustrate the direction of travel but should not be treated as completed evidence of effectiveness until outcome data are available [41].

Successful clinical implementation will therefore depend not only on algorithmic performance but also on seamless integration into established rehabilitation pathways, multidisciplinary clinical workflows, and patient-centred models of care. Future AI systems should therefore be evaluated not only for predictive accuracy but also for usability, acceptability, feasibility, and their ability to support long-term engagement with physical activity programmes.

Recent technology-based studies outside MS further illustrate the broader movement toward sensor-supported and digitally mediated rehabilitation. For example, VR-based interventions have been evaluated for cognitive outcomes in older adults with mild cognitive impairment, and sEMG-derived features have been used to detect muscle fatigue in community-dwelling older adults [42,43]. These studies do not meet the eligibility criteria for the present MS-focused AI review and were not included among the 21 synthesized studies, but they provide contextual evidence that digital and sensor-based methods are increasingly used to monitor function, fatigue, and rehabilitation outcomes across neurological and ageing populations.

### 4.4. Performance Metrics, Validation, Heterogeneity, and Reproducibility

The performance metrics reported across studies were heterogeneous and not directly comparable. Depending on the modelling task, studies reported or implied classification or prediction metrics such as accuracy, sensitivity, specificity, area under the curve, precision, recall, F1 score, estimation error, explained variance, or related indicators. However, differences in target outcomes, sample size, sensor type, feature extraction, validation approach, and population characteristics limit direct comparison. Not all studies reported calibration, uncertainty, decision thresholds, class imbalance handling, explainability, or clinically interpretable error margins. This limits the ability to determine whether apparently strong model performance would translate into reliable clinical decision support in real-world MS rehabilitation or activity monitoring.

Relatively few studies reported calibration performance, decision analytic measures, or clinically meaningful thresholds for model implementation, making it difficult to judge the practical reliability and clinical utility of AI-generated predictions beyond conventional measures of discrimination. Heterogeneity was a major barrier to synthesis. The included studies differed in MS phenotype, disability level, age profile, sample size, sensor technology, data collection environment, feature engineering, algorithm type, target outcome, validation strategy, and reporting detail. Several models were developed in relatively small or controlled datasets, increasing the risk of overfitting and limiting confidence in generalizability.

Such methodological heterogeneity also limits reproducibility and complicates direct benchmarking of AI models developed by different research groups. Future studies should prioritize external validation, prospective testing, transparent reporting of preprocessing and feature selection, reproducible code or model descriptions where feasible, and evaluation across devices, clinical sites, disability levels, and real-world environments. Greater methodological standardization, together with harmonized reporting of performance metrics and validation procedures, would substantially improve comparability between studies and facilitate future evidence synthesis.

### 4.5. Clinical Translation and Future AI-Supported Applications

The most promising future applications are human-supervised rather than autonomous. First, AI could support fatigue-aware activity planning by identifying days or periods when activity progression appears safe, when pacing is advisable, or when rest and symptom management should take priority. Second, AI could support fall-risk-informed exercise adaptation by adjusting balance, gait, and strength tasks according to wearable-derived gait instability, postural sway, daily life turning, recent falls, fear of falling, or deterioration in mobility indicators. Third, AI could identify early disengagement from physical activity programmes by detecting declining step counts, reduced active minutes, worsening fatigue, increasing fall risk, or reduced interaction with digital tools.

These applications should be viewed as complementary clinical decision support functions designed to assist rehabilitation professionals rather than to replace individualized clinical assessment or shared decision-making. Their potential value lies in enabling timelier, data-informed, and personalized rehabilitation strategies while maintaining clinician oversight throughout the care pathway.

AI could enable just-in-time adaptive interventions by delivering prompts, coaching, or feedback when patients are most likely to benefit and least likely to be harmed by overexertion [44]. In MS, such systems must be cautious, as poorly timed prompts could worsen fatigue, frustration, or unsafe activity. AI may also support clinician dashboards that combine wearable data, smartphone data, patient-reported outcomes, and rehabilitation goals into interpretable summaries. Such dashboards should augment clinical reasoning, and shared decision-making, not replace professional judgement.

Future clinical implementation should therefore preserve meaningful human oversight, ensuring that AI-generated recommendations remain clinically appropriate, individualized, transparent, and aligned with patient preferences, rehabilitation goals, and changing disease status.

Future AI-supported MS rehabilitation may also be influenced by emerging paradigms such as explainable AI, federated learning, multimodal AI, and foundation models. Explainable AI may help clinicians and patients understand why a model predicts fatigue, fall risk, fear of falling, reduced mobility, or low physical activity. Federated learning could allow models to be trained across institutions without centralizing sensitive patient data [45]. Multimodal AI may integrate clinical measures, wearable and smartphone data, patient-reported outcomes, and neuroimaging biomarkers. In the longer term, combining digital mobility biomarkers with structural MRI, diffusion MRI, functional MRI, and connectomics could support more precise models of disability progression, fatigue, mobility decline, and rehabilitation response in MS. Generalist or foundation medical AI models may also influence future research, although their use in MS rehabilitation will require careful validation, governance, and clinical oversight [46].

Explainable AI may additionally enhance clinician trust, facilitate patient acceptance, and support regulatory approval by improving the transparency and interpretability of AI-assisted rehabilitation systems. Similarly, multimodal AI has the potential to capture the multidimensional nature of MS by integrating functional, behavioural, clinical, and neuroimaging information into more comprehensive models of disease status and rehabilitation needs.

### 4.6. Reporting, Ethics, Governance, and Digital Health Equity

AI applications in MS require transparent reporting, external validation, and appropriate clinical governance. Prediction models should clearly report datasets, preprocessing, missing data handling, feature selection, model architecture, validation strategy, calibration, uncertainty, and performance metrics, in line with TRIPOD + AI guidance for prediction models using regression or machine learning methods [47]. If AI-supported physical activity or rehabilitation interventions are evaluated in clinical trials or trial protocols, CONSORT-AI and SPIRIT-AI provide relevant reporting extensions for trial reports and protocols involving AI interventions [48,49]. For early-stage clinical evaluation of AI-driven decision support systems, DECIDE-AI may further support structured and transparent assessment before large-scale implementation [50].

Adherence to these reporting frameworks will improve methodological transparency, facilitate independent replication, and enhance confidence in the clinical applicability of AI-based rehabilitation research. Standardized reporting is particularly important in rapidly evolving fields, where differences in study design and analytical methods may otherwise hinder comparison across investigations.

Ethical and regulatory considerations are equally important. AI tools may collect sensitive data on mobility, fatigue, sleep, location, behaviour, and daily functioning, falls and rehabilitation adherence. Therefore, privacy, consent, explainability, equity, accessibility, data governance, and patient burden should be addressed from the earliest stages of development and evaluation [51,52].

Algorithmic transparency is particularly important when AI outputs may influence activity recommendations, pacing advice, fall risk interpretation, fear-of-falling management, or rehabilitation decisions. Patients and clinicians should be able to understand the basis, limitations, and uncertainty of AI-generated outputs.

Beyond technical performance, successful implementation will also depend on maintaining patient trust, clinician confidence, and regulatory compliance throughout the AI lifecycle, from model development and validation to deployment and post-implementation monitoring.

Digital health equity also requires specific attention. AI-supported monitoring may depend on access to smartphones, wearable devices, stable internet connectivity, digital literacy, and the ability to use devices consistently. People with MS may face cognitive, visual, dexterity, mobility, fatigue-related, socioeconomic, or geographic barriers that reduce access to digital monitoring and telerehabilitation. If these barriers are not addressed, AI systems could widen rather than reduce disparities in rehabilitation access. Human factors, usability, affordability, and accessibility should therefore be treated as core clinical and implementation requirements, not optional design features, especially when AI-driven systems aim to support physical activity and behaviour change [53].

Future studies should also evaluate algorithmic fairness across clinically relevant demographic and disease-related subgroups, including age, sex, disability level, MS phenotype, socioeconomic status, and digital access, to ensure that AI-assisted rehabilitation tools perform equitably across diverse patient populations. Consideration should also be given to co-design approaches involving people with MS, caregivers, rehabilitation professionals, and other stakeholders during the development and evaluation of AI-supported systems. Such collaborative approaches may improve usability, accessibility, clinical relevance, and long-term adoption in routine practice.

### 4.7. Strengths and Limitations

This review demonstrates several strengths. A clearly defined AI-only eligibility framework was applied; general digital health studies were not considered AI studies unless they included explicit AI/ML or model-derived predictive analytics; both biomedical and technology-oriented sources were included; and Scopus/Web of Science searches strengthened coverage of biomedical engineering, gait analysis, and sensor fusion literature. The inclusion of 21 studies provides a broader and more reliable map of AI activity-related research in MS. The review also distinguishes between evidence focused on prediction or monitoring and evidence from completed AI-guided interventions. As a result, the synthesis avoids overstating the current maturity of the field.

An additional strength of this review is the structured methodological characterization of AI models, including learning strategies, validation approaches, performance reporting, and methodological limitations. This provides a more comprehensive assessment of AI maturity and translational readiness than reviews focusing solely on clinical outcomes or digital health technologies.

It is important to acknowledge the limitations of this review. The included studies were heterogeneous in sample, technology, data source, AI method, validation strategy, and outcome. Several studies used laboratory or controlled settings, and some technology-oriented papers provided less complete clinical reporting than biomedical clinical trials. This scoping review did not perform a formal risk-of-bias assessment or meta-analysis. This approach is consistent with the mapping objectives of a scoping review, but it weakens conclusions about effectiveness. Inter-rater agreement was not calculated during the screening process. Publication bias is possible, because studies reporting unsuccessful models, weak predictive performance, or limited feasibility may be less likely to be published in rapidly developing technology-oriented fields. Furthermore, because AI research evolves rapidly, additional relevant studies may have been published after completion of the literature search. Model generalizability remains a major challenge. AI models developed using specific MS cohorts, sensor devices, data collection protocols, laboratory settings, smartphone platforms, or definitions of fall risk may not be directly transportable to other populations or clinical environments. Performance may vary across relapsing-remitting and progressive MS, disability levels, age groups, assistive device use, comorbidities, rehabilitation settings, and wearable device types. Future studies should evaluate external validation, device independence, calibration, subgroup performance, and clinical utility before AI-supported mobility or physical activity tools are considered for routine implementation.

Algorithmic fairness and dataset representativeness are also unresolved. Most included studies used relatively small datasets, and reporting of MS phenotype, disability level, age distribution, sex, ethnicity, socioeconomic status, comorbidities, assistive device use, and digital access was variable. It remains unclear whether AI models trained in these datasets would perform equally well across younger and older adults, mild and advanced disability, different MS phenotypes, or patients with different access to wearable and smartphone-based monitoring. Future studies should report subgroup composition transparently and evaluate model performance across clinically relevant strata.

Future research should also prioritize multicenter collaborations, standardized reporting practices, open and reproducible validation frameworks where feasible, and clinically meaningful outcome measures to accelerate the safe translation of AI technologies into routine MS rehabilitation.

## 5. Conclusions

This scoping review identified 21 original AI studies relevant to physical activity, gait, mobility, fatigue, fall risk, and real-world functioning in people with MS. The evidence base indicates that AI can extract clinically relevant patterns from gait, postural sway, wearable, smartphone, connected device, chair stand, daily life turning, clinical, and patient-reported data. However, current evidence does not yet demonstrate that AI-supported systems improve physical activity, adherence, fatigue burden, fall risk, health-related quality of life, or functional independence in people with MS. The field should therefore be interpreted as promising but still early, with AI currently more mature as a monitoring, classification, and prediction tool than as a validated intervention.

The next step is prospective, externally validated, human-supervised, fatigue-aware, and safety-sensitive research testing whether AI-informed feedback can improve patient-centred outcomes in real-world MS care. If supported by robust prospective validation, transparent reporting, appropriate clinical governance, and meaningful human oversight, AI has the potential to become an integral component of personalized physical activity and rehabilitation programmes for people with MS.

## Figures and Tables

**Table 1:** Included original AI studies in chronological order, with main contributions and limitations.

Study	Clinical Application	Sample/Data Source	AI/ML Approach	Learning Type	Validation/Performance Reporting	Main AI Methodological Limitation
Gong et al., 2016 [17]	Gait assessment; mobility characterization.	Inertial body sensor gait data in people with MS.	Deep convolutional neural networks applied to temporal and spectral inertial sensor patterns.	Supervised deep learning	Accuracy/sensitivity/specificity/AUC or error metrics variably reported; external validation uncommon or not clearly reported.	Conference paper; limited reporting compared with full clinical studies; external validation details limited.
McGinnis et al., 2017 [18]	Gait speed; mobility monitoring.	Skin-mounted wearable sensor data from healthy controls and people with MS.	Machine learning gait speed estimation from wearable sensor features.	Supervised ML classification	Accuracy/sensitivity/specificity/AUC or error metrics variably reported; external validation uncommon or not clearly reported.	Primarily estimation/validation work; clinical thresholds and generalizability across devices require further study.
Tong et al., 2019 [19]	Fatigue and health state monitoring relevant to activity planning.	198 people with MS using connected wellness devices for approximately 6 months.	Ensemble regression and personalized adaptation to predict Fatigue Severity Scale and EQ-5D scores.	Supervised predictive modelling	Accuracy/sensitivity/specificity/AUC or error metrics variably reported; external validation uncommon or not clearly reported.	Not an intervention trial; physical activity was not the primary outcome; workflow integration remains uncertain.
Sun et al., 2019 [20]	Fall risk; balance; safe activity.	Postural sway measures in people with MS.	Machine learning prediction of fall risk from postural sway features.	Supervised predictive modelling	Accuracy/sensitivity/specificity/AUC or error metrics variably reported; external validation uncommon or not clearly reported.	Prediction study; prospective external validation and clinical actionability remain limited.
Meyer et al., 2021 [21]	Fall risk; safe mobility; activity safety.	Wearable sensor-derived gait data in people with MS.	Deep learning classification of fall risk from gait.	Supervised deep learning	Accuracy/sensitivity/specificity/AUC or error metrics variably reported; external validation uncommon or not clearly reported.	Fall risk classification does not directly prove increased physical activity; external prospective validation is needed.
Creagh et al., 2021 [22]	Ambulation; remote mobility monitoring.	Smartphone-based remote ambulation data.	Interpretable deep learning models for remote characterization of ambulation.	Supervised deep learning	Accuracy/sensitivity/specificity/AUC or error metrics variably reported; external validation uncommon or not clearly reported.	Monitoring-oriented; not designed to test AI-guided behaviour change or exercise prescription.
Kaur et al., 2021 [23]	Gait dynamics; MS mobility impairment.	Instrumented treadmill gait data from 20 people with MS and 20 matched controls.	ML classification using spatiotemporal and kinetic gait dynamics with normalization strategies.	Supervised ML classification	Accuracy/sensitivity/specificity/AUC or error metrics variably reported; external validation uncommon or not clearly reported.	Small cohort; laboratory treadmill setting; classification of MS status rather than direct physical activity improvement.
Piryonesi et al., 2021 [24]	Falls; fall-related injuries; risk stratification.	Clinical and fall/injury-related data in people with MS.	Machine learning algorithms for predicting falls and injuries.	Supervised predictive modelling	Accuracy/sensitivity/specificity/AUC or error metrics variably reported; external validation uncommon or not clearly reported.	Model performance depends on available variables and dataset representativeness; prospective clinical validation is required.
Trentzsch et al., 2021 [25]	Subtle gait change; mobility monitoring.	Standardized gait analysis data in people with MS.	Machine learning algorithms for identifying gait parameters sensitive to subtle MS-related change.	Supervised ML classification	Accuracy/sensitivity/specificity/AUC or error metrics variably reported; external validation uncommon or not clearly reported.	Clinical meaning of subtle model-selected parameters requires longitudinal validation.
Hu et al., 2022 [26]	Gait impairment; walking assessment.	Raw data from an instrumented walkway in people with MS.	ML classification of MS patients based on walkway-derived gait data.	Supervised ML classification	Accuracy/sensitivity/specificity/AUC or error metrics variably reported; external validation uncommon or not clearly reported.	Laboratory-based; not a free-living physical activity intervention; clinical decision thresholds need validation.
Hu et al., 2022 [27]	Walking difficulty; balance; patient-reported function.	Instrumented gait and balance-related data linked to subjective walking/balance difficulty.	ML models used to corroborate subjective ratings.	Supervised ML classification	Accuracy/sensitivity/specificity/AUC or error metrics variably reported; external validation uncommon or not clearly reported.	Cross-sectional/modelling orientation; does not establish causality or improvement with AI-guided intervention.
Tulipani et al., 2022 [28]	Fall status; functional mobility; chair stand performance.	Unsupervised 30 s chair stand performance assessed by wearable sensors in MS.	Sensor-based prediction of fall status from functional test performance.	Supervised predictive modelling	Accuracy/sensitivity/specificity/AUC or error metrics variably reported; external validation uncommon or not clearly reported.	Eligibility depends on model-derived prediction; external validation and clinical deployment evidence remain limited.
Arpan et al., 2022 [29]	Daily life mobility; turning; fall prediction.	Instrumented daily life gait and turning measures in people with MS.	Predictive modelling of falls from real-world gait and turning features.	Supervised predictive modelling	Accuracy/sensitivity/specificity/AUC or error metrics variably reported; external validation uncommon or not clearly reported.	Prediction rather than intervention; device-specific and context-specific transportability require study.
Schumann et al., 2022 [30]	Gait analysis; fall risk detection.	Standardized gait analysis data in people with MS.	Feature selection ensemble and machine learning algorithms for fall risk detection.	Supervised ML classification	Accuracy/sensitivity/specificity/AUC or error metrics variably reported; external validation uncommon or not clearly reported.	Potential risk of overfitting and limited generalizability without external validation.
Meyer et al., 2022 [31]	Walking bouts; fall risk classification; dataset methodology.	Open-source wearable gait dataset in persons with MS.	Fall risk classification performance evaluated across walking bout durations.	Supervised ML classification	Accuracy/sensitivity/specificity/AUC or error metrics variably reported; external validation uncommon or not clearly reported.	Dataset and classification-context-dependent; performance may not transport across devices or settings.
Gül et al., 2023 [32]	Walking parameter classification; differential motor profiles.	Walking parameters in individuals with MS and Parkinson’s disease.	Machine learning discrimination using walking parameters.	Supervised ML classification	Accuracy/sensitivity/specificity/AUC or error metrics variably reported; external validation uncommon or not clearly reported.	Comparative disease classification focus; less directly tied to activity promotion or fall prevention.
Moebus et al., 2024 [33]	Fatigue prediction; activity pacing.	Wearable sensor data from people with MS and healthy controls.	Digital biomarker modelling for predicting daily fatigue.	Supervised predictive modelling	Accuracy/sensitivity/specificity/AUC or error metrics variably reported; external validation uncommon or not clearly reported.	Prediction accuracy and patient-level actionability require further prospective intervention testing.
Gashi et al., 2024 [34]	Real-world monitoring; disability; fatigue; function.	Mobile and wearable sensor data from people with MS and controls.	ML modelling of MS status, MS type, disability and fatigue.	Supervised ML classification	Accuracy/sensitivity/specificity/AUC or error metrics variably reported; external validation uncommon or not clearly reported.	Complex sensor ecosystem; translation into routine care requires validation, usability, and governance.
**Özgür et al., 2024 [35]**	**Fall risk factor; risk stratification.**	**Clinical and fall risk factor data in people with MS.**	**Machine learning approach to determine fall risk factor.**	**Supervised ML classification**	**Accuracy/sensitivity/specificity/AUC or error metrics variably reported; external validation uncommon or not clearly reported.**	**Risk factor modelling may not directly translate into intervention without prospective testing and subgroup validation.**
**Schumann et al., 2024 [36]**	**Fear of falling; gait; activity avoidance.**	**Standardized gait analysis data in people with MS.**	**Machine learning algorithms to detect fear of falling.**	**Supervised ML classification**	**Accuracy/sensitivity/specificity/AUC or error metrics variably reported; external validation uncommon or not clearly reported.**	**Outcome is behavioural/perceptual and requires validation against longitudinal activity and fall outcomes.**
Monaghan et al., 2025 [37]	Real-world physical activity.	Clinical, sensor-based, and self-reported measures in people with MS.	Integrated predictive models for real-world physical activity.	Supervised predictive modelling	Accuracy/sensitivity/specificity/AUC or error metrics variably reported; external validation uncommon or not clearly reported.	Prediction study rather than AI-guided intervention; needs prospective testing of whether predictions improve activity outcomes.

**Table 2:** Structured synthesis of AI applications, data sources, validation issues, and translational maturity.

Clinical Application Area	Included Studies	Main Data Sources	Main AI/ML Approaches	Reported Performance/Validation Pattern	Translational Maturity and Main Limitations
**Gait analysis and mobility classification**	[17,18,22,23,25,26,27,32]	Inertial body sensors, skin-mounted wearable sensors, smartphones, instrumented treadmill data, instrumented walkways, standardized gait analysis systems.	Deep learning, supervised ML classification, gait speed estimation, feature-based gait modelling.	Mostly internal or study-specific validation; performance metrics variably reported across studies.	Useful for objective mobility phenotyping, but mainly laboratory- or device-specific; external validation and clinical thresholds remain limited.
**Fall risk and fall status prediction**	[20,21,24,28,29,30,31,35,36]	Postural sway, wearable gait data, clinical fall/injury data, chair stand tests, daily life gait and turning signals, standardized gait analysis.	Supervised ML, deep learning, predictive modelling, feature selection ensembles.	Classification metrics such as accuracy, sensitivity, specificity, AUC, or related measures were reported inconsistently; external prospective validation was uncommon.	Largest and clinically important domain, but most models remain predictive rather than interventional; risk of overfitting, limited subgroup validation, and uncertain clinical actionability.
**Fatigue and health state prediction**	[19,33,34]	Connected wellness devices, wearable sensors, mobile and wearable sensor data, patient-reported fatigue and health state measures.	Ensemble regression, digital biomarker modelling, mobile/wearable ML modelling.	Prediction error or model performance metrics reported, but calibration, patient-level actionability, and clinical workflow testing remain limited.	Highly relevant for fatigue-aware activity planning, but evidence does not yet show that AI-guided feedback reduces fatigue burden or improves activity adherence.
**Ambulation monitoring and remote functional assessment**	[22,26,27,34]	Smartphone ambulation data, instrumented walkway data, gait/balance data, mobile and wearable sensor streams.	Interpretable deep learning, supervised ML classification, mobile/wearable modelling.	Some studies emphasized interpretability, but broader explainability, calibration, fairness, and external replication were sparsely reported.	Promising for remote monitoring and digital mobility phenotyping, but not yet validated as a routine clinical decision support tool.
**Real-world physical activity prediction**	[37]	Clinical, sensor-based, and self-reported measures.	Integrated predictive modelling.	Prediction performance reported, but interventional impact not tested.	Most directly aligned with physical activity promotion, but remain predictive; prospective trials are needed to determine whether predictions improve activity, adherence, quality of life, or independence.
**Cross-cutting methodological issues**	Across included studies [17,18,19,20,21,22,23,24,25,26,27,28,29,30,31,32,33,34,35,36,37]	Sensor-derived, clinical, patient-reported, and connected device data.	Supervised ML predominated; fewer studies used deep learning, multimodal AI, or advanced predictive frameworks.	Performance reporting was heterogeneous; external validation, calibration, uncertainty, class imbalance handling, and reproducibility were limited.	

## Data Availability

No new primary, patient-level, or individual participant data were created or analyzed in this study. All information was extracted from publicly available published studies cited in the manuscript. The search strategy, PRISMA-ScR flow diagram, exclusion reasons, and included study list are reported in the manuscript and Appendices. Search exports and screening logs are retained by the author team and can be made available to the Editorial Office upon reasonable request, if required.

## Appendix A. PRISMA-ScR Flow Values and Exclusion Reasons

The PRISMA-ScR selection process was conducted within a single eligibility framework. All records and reports, irrespective of source, were screened and assessed using the same predefined inclusion and exclusion criteria. Records were not preferentially selected according to database source, indexing status, publication venue, or disciplinary origin. The final search framework included biomedical, rehabilitation, technology-oriented, trial registry, and citation searching.

**Table A1:** PRISMA-ScR flow values.

Flow Item	Value
Records/reports identified across all sources	332
Duplicates removed	127
Records/reports screened by title and abstract	205
Records/reports excluded at title/abstract screening	160
Full-text reports assessed for eligibility	45
Full-text reports excluded with reasons	24
Original AI studies included in final synthesis	21

Note: Source-level search counts are documented separately in the search log. All records/reports were assessed using the same predefined eligibility criteria. The revised flow values reflect the expanded multisource search, including Scopus and Web of Science.

**Table A2:** Full-text exclusion reasons.

Reason for Full-Text Exclusion	n
Review, systematic review, scoping review, meta-analysis, or protocol without completed outcome data.	8
No explicit AI/ML/deep learning/predictive analytics component.	4
No MS-specific population or extractable MS-specific data.	2
Not directly relevant to physical activity, gait, mobility, fatigue, fall risk, rehabilitation, or real-world functioning.	5
Digital, wearable, telerehabilitation, or remote monitoring study without an original AI/ML or predictive model.	3
Insufficient information, abstract-only record, or duplicate report of an included study.	2
Total full-text reports excluded	24

## Appendix B. Included Studies Verification Table

**Chronological Order**	**Study**	**Year**	**Identifier(s)**	**Reason for Inclusion**
1	Gong et al.	2016	Ref. [17]; doi:10.1109/WH.2016.7764572	Deep learning inertial sensor gait assessment in MS.
2	McGinnis et al.	2017	Ref. [18]; doi:10.1371/journal.pone.0178366	Machine learning gait speed estimation using skin-mounted wearable sensors in MS-relevant mobility assessment.
3	Tong et al.	2019	Ref. [19]; doi:10.1145/3351264	ML prediction of fatigue and health state from connected devices in MS.
4	Sun et al.	2019	Ref. [20]; doi:10.1038/s41598-019-52697-2	Machine learning fall risk prediction using postural sway measures in people with MS.
5	Meyer et al.	2021	Ref. [21]; PMID:32946403; doi:10.1109/JBHI.2020.3025049	Wearables and deep learning for fall risk classification from gait in MS.
6	Creagh et al.	2021	Ref. [22]; PMID:34253769; doi:10.1038/s41598-021-92776-x	Interpretable deep learning for smartphone-based ambulation characterization.
7	Kaur et al.	2021	Ref. [23]; PMID:33378257; doi:10.1109/TBME.2020.3048142	ML gait dynamics classification using an instrumented treadmill.
8	Piryonesi et al.	2021	Ref. [24]; doi:10.1016/j.msard.2021.102740	Machine learning prediction of falls and injuries in people with MS.
9	Trentzsch et al.	2021	Ref. [25]; Brain Sci. 2021, 11, 1078	Machine learning identification of gait parameters suitable for detecting subtle MS-related gait changes.
10	Hu et al.	2022	Ref. [26]; PMID:35354470; doi:10.1186/s12938-022-00992-x	ML classification of MS patients using instrumented walkway data.
11	Hu et al.	2022	Ref. [27]; PMID:36248625; doi:10.3389/frai.2022.952312	ML corroboration of subjective walking and balance difficulty.
12	Tulipani et al.	2022	Ref. [28]; Gait Posture 2022, 94, 19–25	Sensor-based prediction of fall status using unsupervised 30 s chair stand performance assessed by wearable sensors.
13	Arpan et al.	2022	Ref. [29]; PMID: 36015700; PMCID: PMC9415310	Fall prediction based on instrumented daily life gait and turning measures in people with MS.
14	Schumann et al.	2022	Ref. [30]; PMID: 36358403; PMCID: PMC9688245.	Feature selection ensemble and machine learning detection of fall risk using gait analysis in MS.
15	Meyer et al.	2022	Ref. [31]; doi:10.1371/journal.pdig.0000120	Open-source dataset analysis showing the influence of walking bout duration on fall risk classification performance in MS.
16	Gül et al.	2023	Ref. [32]; Turk. J. Neurol. 2023, 29, 277–281	Machine learning discrimination of MS-related walking parameter patterns in comparison with Parkinson’s disease.
17	Moebus et al.	2024	Ref. [33]; PMID:38362266; doi:10.1016/j.isci.2024.108965	Wearable digital biomarkers for daily fatigue prediction.
18	Gashi et al.	2024	Ref. [34]; PMID:38467710; doi:10.1038/s41746-024-01025-8	Mobile/wearable sensor modelling of MS status, type, disability and fatigue.
19	Özgür et al.	2024	Ref. [35]; doi: 10.1186/s12911-024-02621-0. PMID: 39080657; PMCID: PMC11289943.	Machine learning approach to determine fall risk factor in people with MS.
20	Schumann et al.	2024	Ref. [36]; doi: 10.1016/j.msard.2024.105721. Epub 2024 Jun 10. PMID: 38885599.	Machine learning detection of fear of falling using standardized gait analysis in people with MS.
21	Monaghan et al.	2025	Ref. [37]; PMID:40292926; doi:10.3390/s25061780	Prediction of real-world physical activity in MS using clinical, sensor-based and self-reported measures.
